# DNA copy number alterations in pleomorphic leiomyosarcoma: A case report

**DOI:** 10.3892/ol.2014.2030

**Published:** 2014-04-03

**Authors:** MASAHIKO KANAMORI, TAKETOSHI YASUDA, SHIGEHARU NOGAMI, KAYO SUZUKI, TAKESHI HORI

**Affiliations:** 1 Department of Human Science, University of Toyama, Toyama City, Toyama 930-0194, Japan; 2 Department of Orthopaedics, University of Toyama, Toyama City, Toyama 930-0194, Japan

**Keywords:** leiomyosarcoma, comparative genomic hybridization, DNA, chromosome

## Abstract

Pleomorphic leiomyosarcoma (P-LMS) is a rare morphological variant of LMS. The current study presents the cytogenetic data of a P-LMS that arose in the axillary region of a 31-year-old male. The results of array-based comparative genomic hybridization for the primary tumor showed DNA copy number alteration (DCNA) gains of 8ptel, 17ptel and 17q11.2 and losses of 2ptel, 7ptel, 7qtel, 10p15, 12p12-13.1, 13q14.2-14.3, 15q25-26 and Yq11. However, a metastatic lesion showed cytogenetic data different from the primary tumor DCNAs, with only the locus of 17ptel (*282M15*/*SP6*) in common between them. These observations add to the spectrum of DCNAs that have been reported in previous cases of LMS and provide novel cytogenetic data.

## Introduction

Previous cytogenetic studies have revealed that complex genetic changes occur in leiomyosarcoma (LMS). Numerical aberrations and structural rearrangements in all chromosomes have been reported in LMS (1,2). No specific primary cytogenetic abnormality characteristics of LMS have been identified, although, consistent chromosomal profiles have been observed and an attempt at a chromosomal classification has been made (1). Recent advances in mapping resolution using array-based comparative genomic hybridization (array CGH) have significantly improved the resolving power in comparison with that of metaphase CGH (3,4). This has provided more information regarding the complexity and exact locations of genomic rearrangements, which result in DNA copy number alterations (DCNAs). Using array CGH, it has previously been reported that an increase in DCNAs is associated with tumor size in LMS (5).

As a morphological variant of LMS, pleomorphic LMS (P-LMS) was initially described as an important differential diagnosis of pleomorphic malignant fibrous histiocytoma by Fletcher in 1992 (6). Subsequently, various studies have examined a series of P-LMS using morphological, immunohistochemical and electron microscopy methods, establishing the occurrence of this variant (7–9). However, cytogenetic and molecular genetic data for P-LMS have not previously been described. Therefore, the current study reports the DCNA observations for a primary tumor and metastatic lymph node in a patient presenting with P-LMS.

## Case report

**Figure 1:**
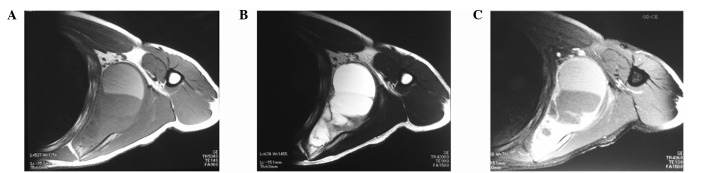
(A) Axial T1-weighted (TR/TE, 534/14) spin echo image of the left axilla showed a cystic tumor with fluid-fluid level, indicative of a soft tissue sarcoma. (B) Axial T2-weighted (TR/TE, 4200/99) spin echo image showed a large cystic tumor. (C) Gadopentetic acid heterogeneously enhanced the mass (TR/TE, 436/13). TR/TE; repetition time/echo time.

A 31-year-old male presented with a mass in the left axilla, which had rapidly enlarged over the previous two months prior to admission to the University Hospital of Toyama (Toyoma, Japan). The patient had an unremarkable clinical history. On examination, a 10×9 cm elastic hard mass, presenting with tenderness, was identified. Neurovascular examination results and laboratory observations were relatively normal. T1- and T2-weighted magnetic resonance imaging showed a large cystic tumor with fluid-fluid levels, indicative of a soft tissue sarcoma (Fig. 1). Angiograms revealed that the tumor was hypervascular.

**Figure 2:**
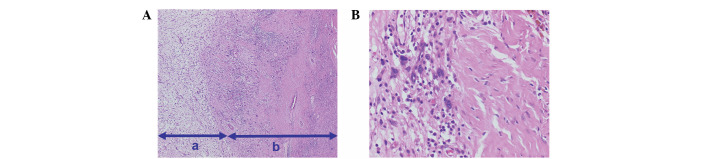
(A) The tumor was composed of (a) myxomatous and (b) pleomorphic areas (H&E; magnification, ×40). (B) The pleomorphic area contained proliferative ovoid or short-spindle atypical cells and a number of mitotic figures were present (H&E; magnification, ×100).

An open tumor biopsy was performed and the pathology was determined as a P-LMS (Fig. 2). Following preoperative chemotherapy for two days with doxorubicin (40 mg/day) and ifosfamide (4 g/day), the tumor was surgically resected. Gross examination of the resected specimen showed an elastic firm and tan-to-yellow mass; its largest diameter was 14 cm. The tumor had a large cyst that contained a brown serous fluid. An enlarged regional lymph node (metastasis) was also resected.

**Figure 3:**
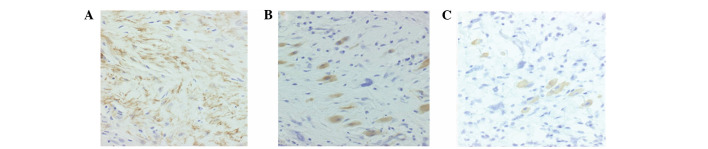
Tumor cells were immunoreactive to (A) smooth muscle actin, (B) h-caldesmon and (C) muscle actin (HHF-35) (immunohistochemical staining; magnification, ×100).

Microscopically, the tumor consisted of myxomatous and pleomorphic areas with proliferating ovoid or short-spindle atypical cells and a number of mitotic figures were observed (Fig. 2). Immunohistochemistry revealed that the tumor cells were positive for vimentin, h-caldesmon and muscle actin (HHF-35; Fig. 3), with slight positivity for smooth muscle actin (SMA). However, these cells were negative for desmin, S-100, cluster of differentiation (CD)31, CD34, CD56, CD99, cytokeratin, c-kit, calretinin and p53 (DakoCytomation, Copenhagen, Denmark).

The tumor specimens were frozen and stored at −80°C until the CGH analysis was conducted. Genomic DNA was isolated from a tumor sample by standard procedures using proteinase K digestion and phenol-chloroform extraction (10,11). Hybridization and analysis by array CGH were performed according to the manufacturer’s instructions (Vysis-Abbott Japan, Inc., Tokyo, Japan). The array CGH comprised of 287 clones that included important tumor suppressors and oncogene loci. Tumor DNA (n=100) was labeled by random priming with fluorescent-labeled cy3-dUTP (Perkin-Elmer, Waltham, MA, USA) and normal reference DNA (n=100) was labeled with cy5-dUTP. The tumor and control DNAs were mixed with Cot-1 DNA (Vysis-Abbott Japan, Inc.), precipitated and resuspended in a microarray hybridization buffer, which contained 50% formamide. The hybridization solution was heated to 80°C for 10 min to denature the DNA and subsequently incubated for 1 h at 37°C. Hybridization was performed for 72 h in a moist chamber, followed by post-hybridization washing in a 50% formamide/2× saline sodium citrate buffer at 45°C. Slides were mounted in phosphate-buffered saline containing 4′,6-diamidino-2-phenylindole (array DAPI solution). Fluorescence intensity images were captured from the hybridized microarray slides using a GenoSensor Reader System equipped with Array 300 Software (Vysis-Abbott Japan, Inc.). The total intensity and intensity ratio of the two dyes for each spot were automatically calculated. The diagnostic cut-off levels representing the gains and losses of the DCNAs were set to an upper threshold of 1.25 and a lower threshold of 0.75.

The current study was approved by the Institutional Review Board for Human Use of the University Hospital of Toyama. Written informed consent for the publication of the patient’s data was obtained from the patient.

**Figure 4:**
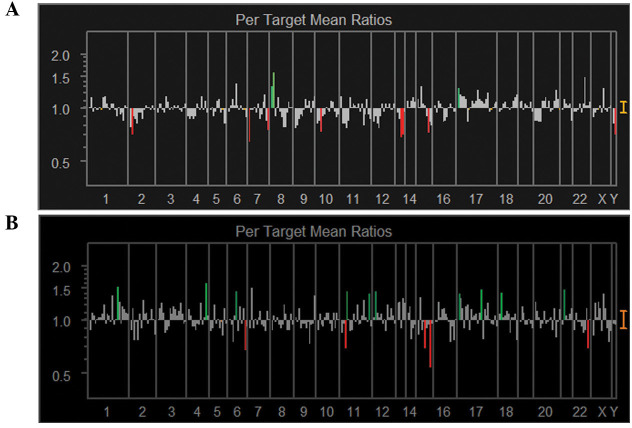
Compared with the primary lesion (A), the genetic aberrations in the metastatic lesion (B) had increased.

The genetic instabilities of the primary tumor and metastatic lymph node were analyzed and compared in the present study. The DCNAs of all chromosomes were determined (Fig. 4). DCNAs that exhibited marked gains (1.25) or losses (0.75) were selected and are listed in Table I. Of the 287 clones represented on array CGH, the primary tumor showed four DCNAs (1.4%) with gains and nine DCNAs (3.1%) with losses. In comparison, the metastatic lymph node showed 11 DCNAs (3.8%) with gains and six DCNAs (2.1%) with losses. Genetic aberrations in the metastatic lesion were increased compared with the primary lesion. Only one DCNA, 17ptel (*282M15/SP6*), was increased in the two samples.

The current study focused on the gain of 8ptel (*D8S504* and *D8S596*) and the loss of 13q14.2-14.3 (*D13S319* and *D13S25*) in the primary tumor, since these loci showed marked changes on the two different arrays for the same locus. These DCNAs may provide various starting points for identifying candidate genes that are associated with oncogenesis. However, these genetic changes were not observed in the metastatic lymph node (Table I).

## Discussion

Analysis of DCNAs to identify the molecular events in soft tissue sarcomas is important. Array CGH technology (3,4) enables the detection of specific genes with DCNAs and may be used to screen for genomic imbalances in human solid cancers. The key biological value of high-resolution array CGH is its ability to detect small amplicons and deletions that potentially harbor specific oncogenes or suppressor genes.

P-LMS is usually defined by an association between areas of undifferentiated pleomorphic sarcoma and areas showing morphological, immunohistochemical or ultrastructural evidence of smooth muscle differentiation. SMA, desmin and HHF-35 are specific to smooth muscle. The novel marker, h-caldesmon, is highly specific for smooth muscle differentiation, however, is expressed in only 40% of cases (9). To date, CGH analysis has not been performed to differentiate LMS subtypes. Therefore, we were unable discuss P-LMS DCNAs from previous reports.

Riva *et al* (12) previously identified a 19p deletion in a single case of recurring LMS. In addition, a previous CGH analysis of 28 cases of LMS (13) reported that a 13q14–q21 loss and 5p14-pter gain at diagnosis may be used to identify patients with LMS who are likely to have reduced survival times. The most frequent losses detected were 10q (20 cases; 69%) and 13q (17 cases; 59%), the most frequent gain that was detected was 17p (16 cases; 55%), and the high-level amplifications that were detected were 17p (seven cases; 24%) and 8q (six cases; 21%). These observations may indicate early changes during LMS tumorigenesis.

The array CGH analysis results of the current study for a primary tumor indicated that the 8ptel (*D8S504* and *D8S596*) gain and 13q14.2-3 (*D13S319* and *D13S25*) loss were target genes. The loss of 13q14.2-3 has been reported previously (13). The most marked changes (1.25 or 0.75) in the metastatic tumor occurred in 17 DCNAs. Among these loci, *WI-5663* (1q21), *SHGC-5557* (12ptel), *282M15/SP6* (17ptel) and *RUNX1* (21q22.3) were observed in our previously reported case of metastatic osteosarcoma (10).

The current study reports the DCNA observations for a primary tumor and metastatic lymph node in a patient with P-LMS. The gain of 8ptel (*D13S319*) and the loss of 13q14.2-14.3 (*D13S25*) may be involved in tumor progression and metastasis. However, further cytogenetic study is required to elucidate the DCNAs in P-LMS.

## Figures and Tables

**Table I:** Summary of DCNA data in the current case of pleomorphic LMS.

A, Gain					

Primary tumor			Lymph node metastasis		
	
Gene name	Locus	DCNAs	Gene name	Locus	DCNAs
*D8S504*	8p tel	1.31	*WI-5663, WI-13414*	1q21	1.54
*D8S596*	8p tel	1.56	*4QTEL11*	4q tel	1.59
*282M15/SP6*	17p tel	1.29	*HTR1B*	6q13	1.44
*NF1 3′*	17q11.2	1.26	*CDKN1C(p57)*	11p15.5	1.45
			*WI-6509*	11q tel	1.39
			*SHGC-5557*	12p tel	1.44
			*SNRPN*	15q12	1.34
			*282M15/SP6*	17p tel	1.39
			*PPARBP(PBP)*	17q12	1.48
			*SHGC17327*	18p tel	1.42
			*RUNX1(AML1)*	21q22.3	1.49

B, Loss					

Primary tumor			Lymph node metastasis		
	
Gene name	Locus	DCNAs	Gene name	Locus	DCNAs

*2PTEL27*	2p tel	0.72	*ESR1*	6q25.1	0.69
*G31341*	7p tel	0.65	*HRAS*	11p15.5	0.71
*7QTEL20*	7q tel	0.76	*MAP2K5*	15q23	0.70
*D10S249,D10S533*	10p15	0.75	*PACE4C*	15q tel	0.55
*CDKN1B(p27)*	12p13.1-p12	0.78	*stSG30213*	16q tel	0.78
*D13S319*	13q14.2	0.69	*ARSA*	22q tel	0.71
*D13S25*	13q14.3	0.72			
*IGF1R*	15q25–q26	0.74			
*AZFa region*	Yq11	0.72			

DCNAs, DNA copy number alterations; LMS, leiomyosarcoma.
